# Molecular Identification of Agents of Human Cutaneous Leishmaniasis and Canine Visceral Leishmaniasis in Different Areas of Iran Using Internal Transcribed Spacer 1 PCR-RFLP

**Published:** 2018-06-13

**Authors:** Aref Teimouri, Mehdi Mohebali, Elham Kazemirad, Homa Hajjaran

**Affiliations:** 1 Department of Medical Parasitology and Mycology, Tehran University of Medical Sciences, Tehran, Iran; 2 Students Scientific Research Center, Tehran University of Medical Sciences, Tehran, Iran; 3 Center for Research of Endemic Parasites of Iran, Tehran University of Medical Sciences, Tehran, Iran

**Keywords:** *Leishmania*, ITS1 gene, PCR-RFLP, Iran

## Abstract

**Background:**

Leishmaniasis is a major medical health problem and distributes in nearly half of 31 provinces of Iran. We aimed to identify cutaneous and visceral *Leishmania* spp. isolated from infected humans and domestic dogs in various regions of Iran, 2010–2013.

**Methods:**

DNA was extracted from 108 lesion exudate samples of suspected patients to cutaneous leishmaniasis and nine liver and spleen aspirates of infected dogs cultured in RPMI-1640 and amplified using partial sequence of ITS1 gene. The PCR amplicons were digested using *Hae*III endonuclease enzyme and used in restriction fragment length polymorphism (RFLP) assay. Then, 48 amplicons representing various hosts were sequenced and compared to sequences from GenBank databases using BLAST.

**Results:**

PCR-RFLP analysis showed that 60 and 48 CL patients were infected by *Leishmania tropica* and *L. major*, respectively. From nine canine visceral leishmaniasis (CVL) isolates, eight isolates were identified as *L. infantum* and one as *L. tropica*. The greatest similarity of 95.7% in ITS1 region was seen between *L. infantum* and *L. major*. Furthermore, the lowest similarity with 65.7% was seen between *L. tropica* and *L. major*. Intra-species comparison of ITS1 region in *L. infantum, L. major* and *L. tropica* isolates were showed 100%, 98.2% and 72.4 % similarities, respectively.

**Conclusion:**

PCR-RFLP based on ITS1 region is an appropriate method to distinguish three *Leishmania* spp. of *L. major*, *L. tropica,* and *L. infantum*. In intra-species comparison of ITS1 region, genotypic variations showed that *L. tropica* isolates were more heterogeneous than *L. major* and *L. infantum* isolates.

## Introduction

Cutaneous (CL) and visceral leishmaniasis (VL) include some of the world most neglected diseases in tropical and subtropical areas with an estimated incidence of 0.6–1.0 and 0.05–0.09 million new cases of CL and VL occur worldwide each year, respectively (1). In Iran, CL is considered as a medical health problem in nearly half of 31 provinces (2). Both epidemiological forms of CL are reported in Iran, anthroponotic cutaneous leishmaniasis (ACL) and zoonotic cutaneous leishmaniasis (ZCL) caused by *Leishmania tropica* and *L. major*, respectively (3). Nearly 20000 CL cases are annually reported and the prevalence of leishmaniasis in provinces of Iran suggestively ranges 1.8–37.9% (2). Furthermore, VL is caused by *L. donovani* complex and *L. infantum* is the main agent of VL in endemic areas of Iran with about 100–300 new cases annually. Infections due to *L. infantum* have been reported in humans, domestic dogs and phlebotomine vectors (4, 5). Human, rodents (mainly great Gerbils (*Rhombomys opimus*)) and domestic canines (*Canis familiaris*) are the main reservoir hosts of ACL, ZCL and VL, respectively (6–8).

Recently, a few cases of viscerotropic leishmaniasis caused by *L. tropica* and *L. major* have been reported in immunocompromised patients in Iran (9, 10). Furthermore, *L. infantum* is involved in sporadic CL in the endemic areas of VL in Northwestern Iran (11). Because of wide clinical diversities of leishmaniasis, various responses of patients to treatment, various reservoir hosts of the parasite and to develop effective control strategies in endemic areas, distinguish between *Leishmania* spp. and find dominant spp. in provinces are critical. Since *Leishmania* spp. are morphologically identical; therefore, species identification using microscopy or culture methods is not easily possible. For species characterization, additional methods must be used. One of these methods, MLEE (multilocus enzyme electrophoresis) analysis, remains the current gold standard but requires mass culture of the parasites which is not simply carried out (12). Relatively, molecular methods are sensitive for the detection of low amounts of the parasite.

Nowadays, molecular based analyses have extensively been used for the identification of *Leishmania* spp. as well as other parasites. Identification and phylogenetic implication with various targets such as kDNA genes and introns are routine (13). Furthermore, the ITS region of rDNA has been used in several studies to resolve taxonomic questions and to determine phylogenetic affinities among closely related *Leishmania* species (14, 15). The ITS1 gene is considered as the most appropriate region and gives the best results in *Leishmania* differentiation in the old world (16). The ITS1 locates between 18S and 5.8S rRNA genes and possesses conservative loci targets for the PCR (17). Additionally, it includes satisfactory polymorphisms to facilitate species identification.

The aim of this study was to achieve a better understanding of the current status of various isolates of *Leishmania* spp. in Iranian provinces using ITS1-PCR-RFLP. Furthermore, molecular findings of this study were used to assess phylogenetic relationships between the isolates.

## Materials and Methods

### Sampling

**Fig. 1.**
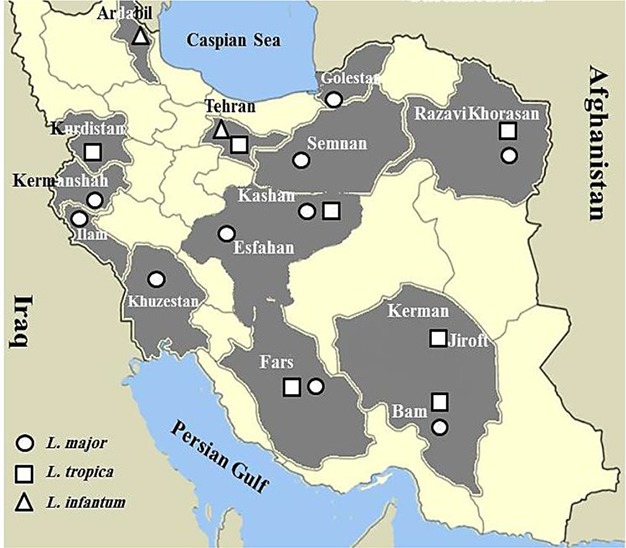
Geographical origins of 148 *Leishmania* spp isolated from cutaneous and visceral leishmaniasis cases identified by ITS1-PCR RFLP (2010–2013)

Samples were collected from 148 lesion exudates of suspected patients to CL referred to Leishmaniasis Laboratory of the School of Public Health, Tehran University of Medical Sciences, Tehran, Iran, and District Health Centers of Iran, 2010–2013. Most of the patients were referred from endemic areas in northeast, southeast, center, west, and southwest of Iran (Fig. 1). Serosity materials of the lesions were smeared on a microscope slide, air-dried, fixed with absolute methanol and stained with Giemsa in 10% phosphate buffer (pH 7.4) for 25min and then examined for amastigotes using light microscope with 1000× magnification. Serosity materials from lesions of CL were cultured in RPMI-1640. Twelve dogs suspected to CVL living in endemic areas were tested serologically using direct agglutination test (DAT). Liver and spleen aspirates of the infected dogs positive for DAT were cultured in RPMI-1640. The parasitological positive dogs showed clinical manifestations of VL including dermatological wounds, ocular variations, weight loss, laziness, lymphadenopathy and splenomegaly (18, 19).

### Ethical approval

The study was approved by the Ethics Committee of the Tehran University of Medical Sciences (Approval No. 13462). No information of the patients was revealed in the study. Verbal informed consents were received from the patients and the dog owners.

### Direct agglutination test (DAT)

Anti *Leishmania* antibodies in dog sera were detected using DAT. Briefly, promastigotes of *L. infantum*, Iranian strain (MCAN/IR/07/Moheb-ghو GenBank Accession No. FJ555210), were cultured in RPMI-1640 with 10% heat-inactivated fetal bovine serum (FBS), trypsinized, fixed with 1.2% formaldehyde (Merck, Germany) and stained with Coomassie brilliant blue (Sigma, USA). Negative and positive controls were used in each experiment. Antibody titers ≥ 1:320 were considered as positive in canines (18).

### Parasite culture and cryopreservation

All samples including lesion exudates of the suspected patients to CL and liver and spleen aspirates of the infected dogs were cultured in RPMI-1640 media supplemented with 10–15% heat-inactivated FBS, 100U/ml of penicillin and 100μg/ml of streptomycin (Gibco, Life technologies, Germany) and incubated at 24–25 °C. Five days after the last sub-culture, parasites were harvested, washed in sterile phosphate buffered saline (PBS, pH 7.2–7.4), stored at −20 °C until use and were preserved in liquid nitrogen for further studies.

### DNA extraction, PCR, and RFLP

Genomic DNA was extracted from pellets of the *Leishmania* cultures using High Pure PCR Template Preparation Kit (Roche Diagnostics, Germany) according to the manufacturer′s instructions. Samples were stored at −20 °C until use. DNA samples from Iranian reference strains of *L. tropica* (MHOM/IR/02/Mash10/Accession No. EF653267), *L. major* (MRHO/IR/11/GOL-2/ Accession No. JN860745) and *L. infantum* (MCAN/IR/ 07/Mash-ir1/ Accession No. EU810776) were used as positive controls. The DNA samples were assessed for the *Leishmania*-specific ribosomal internal transcribed spacer 1 region (ITS1) by PCR amplification using primer pairs of LITSR (F: 5′-CTGGATCATTTTCCGATG-3′) and L5.8S (R: 5′-TGATACCACTTAT CGCACTT-3′). Amplification was carried out using PCR-Ready Premix (Roche, Germany) in a 25μl reaction. The amplification conditions included those described previously (20). PCR products (8μl) were digested with the restriction endonuclease enzyme HaeIII (BsuRI) (Fermentas, Germany) for the species identification according to the manufacturer′s instructions. Amplicons of nearly 300–350bp and restriction fragments were analyzed using 1.5–3% agarose gels containing safe stain, visualized under UV and compared with those from reference strains of *L. tropica*, *L. major* and *L. infantum*.

### Nucleotide sequence and phylogenetic analysis

PCR products from 44 CL and four CVL samples were sequenced using LITSR as forward primers. DNA sequences obtained from the current study were compared to sequences from GenBank database using Basic Local Alignment Search Tool (BLAST). These sequences have been annotated to GenBank and aligned using Clustal X Software and MEGA Software v.6.0 (21). Various statistical methods (maximum likelihood, UPGMA and Neighbor-Joining (NJ) trees) were digitalized for phylogenetic analysis of the aligned sequences. The Tamura 3-parameter option of the neighbor-joining method was shown as the best phylogenetic tree (22). Inter and intra-species similarities of ITS1 region were calculated for *L. major*, *L. infantum* and *L. tropica* isolates using MEGA Software v.6.0. Bootstrap analysis was carried out with 1000 replicates. Accession numbers of 48 *Leishmania* isolates used in this study are recorded in GenBank as follows: JX289844-JX 289881, JN860713-JN860714 and JN860718-JN860725.

## Results

### Microscopic, culture and DAT analysis

Microscopic study demonstrated *Leishmania* amastigotes in 112 out of 148 smears (75.6%) collected from patients suspected to CL. Furthermore, 108 samples (72.9%) were positive for *Leishmania* parasites in culture method. DAT analysis of domestic dog sera showed that 10 sera (83.3%) were positive with titers ≥ 1:320 out of 12 samples. Nine out of 12 dog samples (75.00%) were positive in microscopy and aspirate culture methods.

### *Leishmania* spp identification using RFLP analysis

**Fig. 2.**
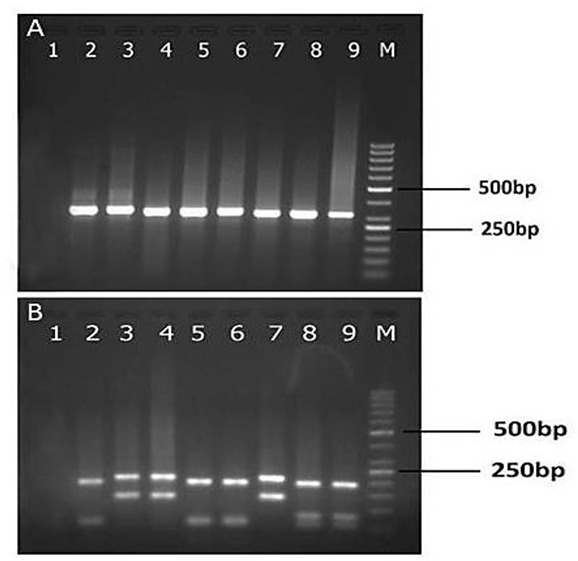
**A)** Electrophoresis of *Leishmania* DNA amplified a 350bp fragment using primers of LITSR and L5.8S. DNA was collected from reference strains and lesion exudates of suspected patients to cutaneous leishmaniasis and liver and spleen aspirates of infected dogs cultured in RPMI-1640. **B)** Digestion patterns of ITS1 amplicons of *Leishmania* spp. from references and patients using *Hae*III enzyme. Line 1, negative control, Line 2, *L. tropica* (Accession Number: EF653267); Line 3, *L. major* (Accession Number: EU810776), Line 8, *L. infantum* (Accession Number: JN860745) as references strain; Line 4–7, patient samples; Line 9, dog sample, M, 50bp ladder

In general, ITS1 PCR was carried out for the diagnosis of *Leishmania* spp, in which positive samples produced amplicons of 300–350bp (Fig. 2A). Digestion of the PCR products with *Hae*III endonuclease produced two bands of 220 and 140bp for *L. major* reference strain. Furthermore, bands of 200, 60 and 5bp and 200, 80 and 40bp were produced for *L. tropica* and *L. infantum* reference strains, respectively (Fig. 2B). Compared to patterns produced in reference strains and CL patients, two profiles were clearly distinguishable as follows: 1) 48 amplicons (44.5%) produced two bands (220 and 140bp) indicating *L. major*, and 2) 60 amplicons (55.5%) produced three bands (200, 60 and 50bp) indicating *L. tropica*. RFLP analysis on dog samples revealed *L. infantum* with three bands of 200, 80 and 40bp in eight cases (88.88%) and *L. tropica* in one case (11.12%).

### Sequencing and phylogenetic tree analysis

**Fig. 3.**
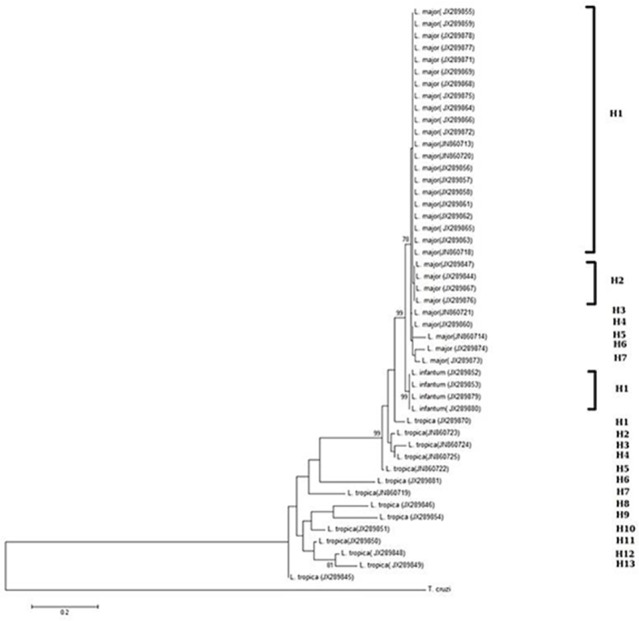
The neighbor-joining tree constructed from ITS1 regions of 48 isolates using Tamura 3-parameter. Numbers above branches correspond to bootstrap valued based on 1000 replicates. Branches without numbers include values of less than 70%. Genbank accession numbers are shown in parentheses

**Fig. 4.**
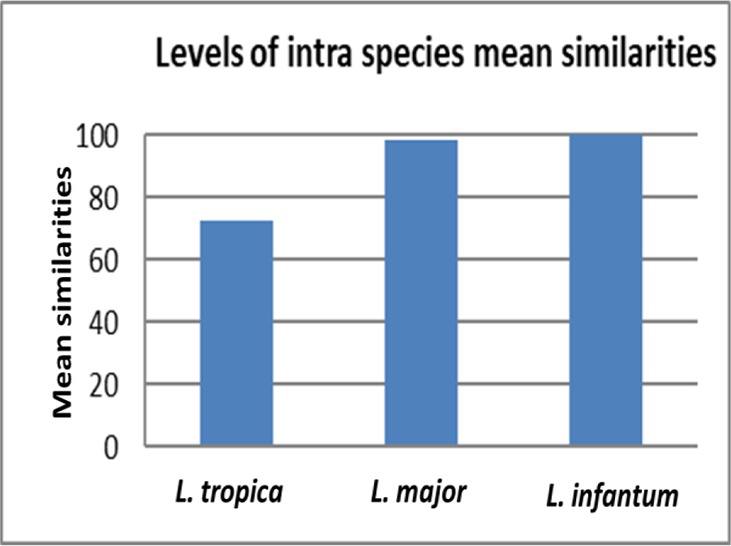
Intra-species mean similarities for *Leishmania* spp. isolated from CL and VL cases based on ITS1 region sequence, 2010–2013

**Fig. 5.**
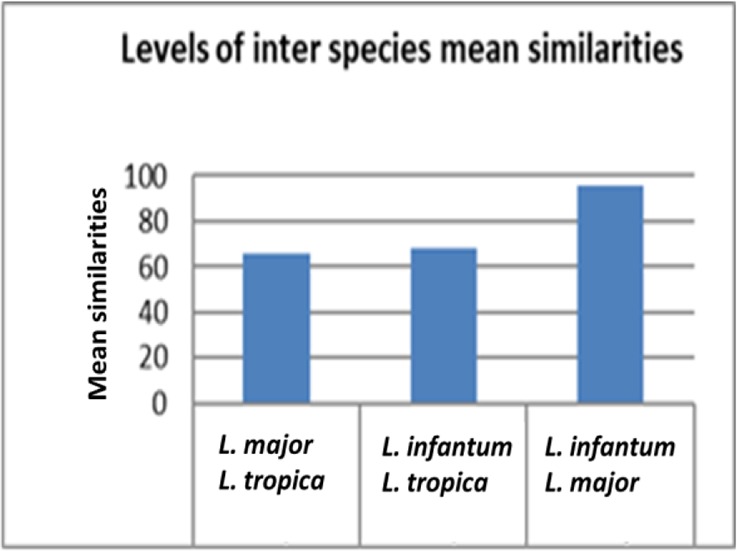
Inter-species mean similarities among *Leishmania* spp. isolated from CL and VL cases based on ITS1 region sequence, 2010–2013

Forty eight amplicons were successfully sequenced. The phylogenetic trees from the sequences of ITS1 fragments clearly showed divergence between *L. major*, *L. infantum*, and *L. tropica*. Furthermore, phylogenetic analysis demonstrated variations by sequencing as 21 haplotypes were shown in 48 Iranian isolates including *L. major* (H1–H7), *L. infantum* formed independent and equal clusters with 94% bootstrap (H1) and *L. tropica* (H1–H13) (Fig. 3). Intra-species similarities of ITS1 region in isolates of *L. infantum*, *L. major*, and *L. tropica* included 100%, 98.2% and 72.4%, respectively (Fig. 4). The highest inter-species similarity of ITS1 region with 95.7% was reported in isolates of *L. infantum* and *L. major* followed by a 68.5% of similarity between *L. infantum* and *L. tropica*. The lowest similarity with 65.7% was reported between *L. tropica* and *L. major* (Fig. 5).

## Discussion

Species identification is critical in diagnosis, treatment and epidemiological studies of *Leishmania* spp. Several studies have amplified ITS-1 region of the ribosomal DNA repeat unit (rDNA-ITS1) for the recognition of Iranian *Leishmania* spp. (8, 23–25). In the present study, a number of unknown *Leishmania* spp. isolated from CL and CVL cases in various endemic regions of Iran was identified by PCR-RFLP and sequencing based on ITS1 region of rRNA gene. In the current study, the major foci of 60 *L. tropica* CL isolates belonged to Khorasan Razavi Province, Bam City (Kerman Province), Kashan City (Isfahan Province). Moreover, one isolate belonged to a CL lupoid patient residing in Shahr-e-Rey in southern Tehran (ACC, JX289854). Forty-eight *L. major* isolates belonged to Golestan, Isfahan, Kermanshah, Khuzestan, Ilam and Semnan Provinces of Iran (Fig. 1). Two isolates recovered from diffuse cutaneous leishmaniasis (DCL) patients in Damghan City of Semnan Province in central (ACC, JN860713) and Dehloran City of Ilam Province in Southwestern Iran (ACC, JN860714) were identified as *L. major*. Of nine CVL isolates, eight isolates were identified as *L. infantum* (six isolates from Ardabil, one isolate from Golestan and one isolate from Tehran provinces) and one isolate as *L. tropica* (from Golestan Province). The phylogenetic analysis revealed 21 haplotypes within the isolates. No correlations were seen between the haplotypes and geographic distribution of the species complexes. In similar studies in Iran, no correlations were demonstrated between the intra-species divergence and geographical distribution based on RAPD-PCR and ITS1 and N-acetylglucosamine-1-phosphate transferase (NAGT) genes with PCR-RFLP methods (3, 26). In the phylogenetic analysis, *L. tropica* isolates showed 13 haplotypes (H1–H13) indicating a significant divergence between *L. tropica* isolates from the other two species (Fig. 3). Although the ITS region is one of the best candidates for the differentiation of *Leishmania* at species and strain levels, limited studies have used ITS sequence analysis for *L. tropica* isolates to compare (27–29).

*Leishmania tropica* (a diploid microorganism) is known as a heterogenous species, quite observed in alignment of the PCR product sequences and in low bootstrap frequencies found in the phylogenetic tree (30–32). The existence of at least two alleles for ITS in rDNA of *Leishmania* spp. may be a good explanation for this phenomenon (33). In the current study, sequences from three *L. tropica* isolates were technically unreadable excluded from the phylogenetic analysis. However, unreadable sequences of ITS-rDNA fragments belonged to positive leishmaniasis cases (24). This can prove high heterogeneity or sometimes mixed infections with two or three *Leishmania* spp. (23, 24, 34, 35).

Similar to pilot studies, a high degree of heterogeneity was seen in *L. tropica* in our study (30, 33, 36, 37). In intra-species analysis, a varied heterogeneity was found at various levels in *Leishmania* spp. using ITS-PCR-RFLP analysis. This included a highest to lowest order of *L. tropica* > *L. major* > *L. infantum* with similarities of 72.4%, 98.2% and 100%, respectively. Relatively, *L. donovani*, *L. infantum* and *L. major* were reported as the less and *L. tropica*, *L. turanica* and *L. gerbilli* as the most divergent complexes (38). In 2005, a heterogeneity variation at various levels in Old World *Leishmania* spp. Was reported with a highest to lowest order of *L. tropica* > *L. aethiopica* > *L. major* > *L. donovani* (39). These findings are similar to studies reported that *L. tropica* isolates included the highest divergence in ITS1 genes (26). Similar to a study on *L. major* with 98.2% similarity, results of the current study showed a limited genetic variation with seven haplotypes seen in *L. major* (H1–H7) (25). Four haplotypes of *L. major* were found in a distinct clade in nine rodent isolates in Central Iran (23) and six haplotypes of *L. major* were identified through PCR-RFLP of rodent samples using ITS1 markers (8).

Results of the present study on *L. infantum* isolates showed no diversities while formed independent and equal clusters with the highest bootstrap values (94%) in the phylogenetic analysis. Moreover, heterogeneity of *L. tropica* with various groups was detected in the current phylogenetic analysis while *L. infantum* isolates consisted of only one haplotype. In a similar study in Turkey using an ITS1 based real-time PCR, genotypic variations of *Leishmania* spp. at species and intra-species levels were observed and heterogeneities were found in *L. tropica* isolates while *L. infantum* isolates formed single group (40). In the present study, the lowest and highest inter-species varieties were seen between *L. major* and *L. infantum* and between *L. tropica* and *L. major* with 4.3% and 34.3% variations, respectively. In the tree, *L. major* was more associated to *L. infantum* than *L. tropica* was. These findings were in contrast to those from studies based on NAGT genesو in which, the lowest inter-species similarity of 95.7% were reported between *L. major* and *L. infantum* (3).

## Conclusion

Sequencing results of *Leishmania* spp. isolated from CL and CVL cases showed 93–98% similarities with other annotated sequences in GenBank database. Therefore, PCR-RFLP based on ITS1 region can be suggested as an adequate method to distinguish *L. major*, *L. tropica* and *L. infantum* which are the most prevalent *Leishmania* spp. in Middle East. Furthermore, genotypic variations based on ITS1 region within inter and intra-species of *Leishmania* spp. have shown that *L. tropica* includes more heterogeneity than *L. major* and *L. infantum* do.
